# Establishment of a risk prediction model for bowel necrosis in patients with incarcerated inguinal hernia

**DOI:** 10.1186/s12911-024-02440-3

**Published:** 2024-02-06

**Authors:** Jiajie Zhou, Xiaoming Yuan

**Affiliations:** Department of General Surgery, Huai’an First People’s Hospital, Nanjing Medical University, Huai’an, Jiangsu Province China

**Keywords:** Inguinal hernia, Nomogram, Risk factor, Incarcerated inguinal hernia, Bowel necrosis

## Abstract

**Introduction:**

Incarceration occurred in approximately 5% to 15% of inguinal hernia patients, with around 15% of incarcerated cases progressing to intestinal necrosis, necessitating bowel resection surgery. Patients with intestinal necrosis had significantly higher mortality and complication rates compared to those without necrosis.The primary objective of this study was to design and validate a diagnostic model capable of predicting intestinal necrosis in patients with incarcerated groin hernias.

**Methods:**

We screened the clinical records of patients who underwent emergency surgery for incarcerated inguinal hernia between January 1, 2015, and December 31, 2022. To ensure balanced representation, the enrolled patients were randomly divided into a training set (*n* = 180) and a validation set (*n* = 76) using a 2:1 ratio. Logistic regression analysis was conducted using the rms package in R software, incorporating selected features from the LASSO regression model, to construct a predictive model.

**Results:**

Based on the results of the LASSO regression analysis, a multivariate logistic regression model was developed to establish the predictive model. The predictors included in the model were Abdominal effusion, Hernia Sac Effusion, and Procalcitonin. The area under the receiver operating characteristic (ROC) curve for the nomogram graph in the training set was 0.977 (95% CI = 0.957–0.992). In the validation set, the AUC for the nomogram graph was 0.970. Calibration curve and decision curve analysis (DCA) verified the accuracy and practicability of the nomogram graph in our study.

**Conclusion:**

Bowel necrosis in patients with incarcerated inguinal hernia was influenced by multiple factors. The nomogram predictive model constructed in this study could be utilized to predict and differentiate whether incarcerated inguinal hernia patients were at risk of developing bowel necrosis.

**Supplementary Information:**

The online version contains supplementary material available at 10.1186/s12911-024-02440-3.

## Introduction

In the past, inguinal hernia (IH) referred to the formation of a sac where the peritoneum protruded through a weak point in the inguinal region. Typically, it contained abdominal contents and was commonly treated through surgical intervention. The surgical repair of inguinal hernia was the most prevalent elective procedure worldwide, with an annual surgical volume exceeding 20 million cases [1]. However, there existed significant disparities in inguinal hernia surgical rates across different countries. In the United Kingdom, the surgical rate stood at 10 per 10,000 individuals, whereas in the United States, the surgical rate amounted to 28 per 10,000 individuals [2].

Patients afflicted with inguinal hernia faced the peril of hernia incarceration if timely surgical intervention was not promptly undertaken. Hernia incarceration entailed the acute entrapment of intra-abdominal viscera within the hernial ring, subsequently leading to necrosis, perforation, and intra-abdominal infection. Emergency surgery was typically imperative in such cases, and patients undergoing emergency surgery exhibited higher rates of complications and mortality compared to those undergoing elective procedures. Patients with inguinal hernias in males typically manifested as asymptomatic or with mild symptoms. However, once intestinal incarceration occurred, patients might have exhibited pronounced abdominal pain symptoms. It was noteworthy that the incidence of incarceration in inguinal hernias was generally less than 0.5% [3] with 15% of patients [4] progressing to an incarcerated state, thereby leading to intestinal necrosis, potentially necessitating bowel resection surgery. The incidence of complications and mortality in emergency surgery for incarcerated inguinal hernia stood at 21–39% and 4–5%, respectively [5]. Patients with intestinal necrosis experienced significantly prolonged hospitalization compared to those without necrosis, with increased rates of complications and a mortality rate ranging from 1 to 7% [6]. Therefore, early recognition of risk factors in patients with inguinal hernia accompanied by intestinal necrosis and timely surgical intervention were of paramount importance for their prognosis.

The objective of this study was to identify the risk factors associated with incarcerated inguinal hernia and intestinal necrosis, aiming to assist surgeons in preoperative evaluation of bowel viability and provide high-level evidence to support their clinical decision-making, enabling them to make precise and informed choices.

## Materials and methods

### Patients

**Fig. 1:**
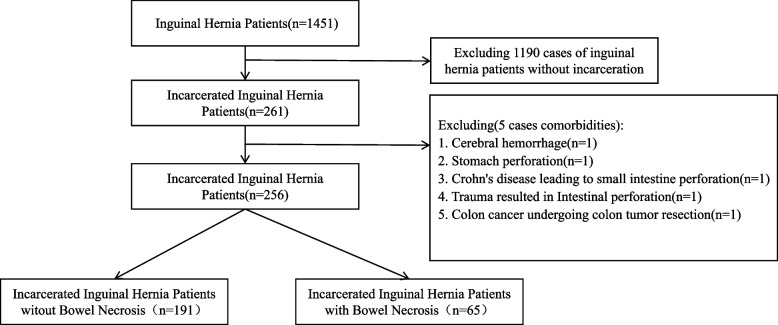
Patients selection protocol

In this retrospective study conducted at a single center, we developed and validated a diagnostic model for predicting intestinal necrosis in patients with incarcerated inguinal hernia (IIH), utilizing clinical data from a Chinese public tertiary hospital. The methodology utilized in this study conformed to the relevant medical guidelines and regulations, and the research acquired approval from the Ethical Committee of Huai’an First People’s Hospital. All participants provided informed consent. Clinical records of patients who underwent emergency surgery for incarcerated inguinal hernia between January 1, 2015, and December 31, 2022, were screened. Included patients had comprehensive clinical data, including blood routine, biochemical indicators, and imaging examination reports. The inclusion and exclusion process is illustrated in the flowchart. A total of 256 patients with incarcerated inguinal hernia were recruited, with 5 patients excluded. Figure 1 delineates the process of screening patients. Inclusion criteria: Patients afflicted with incarcerated inguinal hernias necessitating surgical intervention. Exclusion criterion: Patients necessitating surgical intervention unrelated to an incarcerated hernia. Given the widespread utilization of computed tomography (CT) scanning machines in Chinese computing facilities and the stipulation in the author’s affiliated hospital that all patients with acute abdominal symptoms must undergo radiological examinations preoperatively, encompassing both CT and ultrasound examinations, all patients in this study cohort underwent CT examinations. This practice became a routine procedure in medical institutions in China. Nevertheless, abdominal and inguinal ultrasound examinations sufficed adequately for the purposes of this study.

All included patients underwent surgery, and the occurrence of intestinal necrosis was confirmed. Out of these, 65 patients were diagnosed with intestinal necrosis, while 191 patients were confirmed to be free of intestinal necrosis. To ensure balanced representation, the enrolled patients were randomly divided into a training set (*n* = 180) and a validation set (*n* = 76) using a 2:1 ratio. Baseline characteristics were comparable with no differences between the two groups, as shown in Table 1 [7].

**Table 1:** Baseline characteristics

Characteristic	Total patient (*n* = 256)	IIH witout BN (*n* = 191)	IIH with BN (*n* = 65)	*P*	Validation set (*n* = 76)	Training set (*n* = 180)	*P*
**Gender**				0.005			0.672
Female	60 (23.4%)	36 (18.8%)	24 (36.9%)		16 (21.1%)	44 (24.4%)	
Male	196 (76.6%)	155 (81.2%)	41 (63.1%)		60 (78.9%)	136 (75.6%)	
**Age (years)**				< 0.001			0.664
< 50	71 (27.7%)	65 (34.0%)	6 (9.23%)		23 (30.3%)	48 (26.7%)	
≥ 50	185 (72.3%)	126 (66.0%)	59 (90.8%)		53 (69.7%)	132 (73.3%)	
**Hypertension**				0.697			0.209
No	184 (71.9%)	139 (72.8%)	45 (69.2%)		50 (65.8%)	134 (74.4%)	
Yes	72 (28.1%)	52 (27.2%)	20 (30.8%)		26 (34.2%)	46 (25.6%)	
**Coronary Heart Disease**				0.864			0.525
No	233 (91.0%)	173 (90.6%)	60 (92.3%)		71 (93.4%)	162 (90.0%)	
Yes	23 (8.98%)	18 (9.42%)	5 (7.69%)		5 (6.58%)	18 (10.0%)	
**Diabetes**				0.202			0.564
No	242 (94.5%)	183 (95.8%)	59 (90.8%)		71 (93.4%)	171 (95.0%)	
Yes	14 (5.47%)	8 (4.19%)	6 (9.23%)		5 (6.58%)	9 (5.00%)	
**Cerebral Infarction**				0.087			1.000
No	238 (93.0%)	181 (94.8%)	57 (87.7%)		71 (93.4%)	167 (92.8%)	
Yes	18 (7.03%)	10 (5.24%)	8 (12.3%)		5 (6.58%)	13 (7.22%)	
**Duration of Incarcerated Inguinal Hernia (h)**				< 0.001			0.876
< 12	148 (57.8%)	133 (69.6%)	15 (23.1%)		45 (59.2%)	103 (57.2%)	
≥ 12	108 (42.2%)	58 (30.4%)	50 (76.9%)		31 (40.8%)	77 (42.8%)	
**Location of Incarcerated Inguinal Hernia**				0.913			0.308
Right	162 (63.3%)	120 (62.8%)	42 (64.6%)		44 (57.9%)	118 (65.6%)	
Left	94 (36.7%)	71 (37.2%)	23 (35.4%)		32 (42.1%)	62 (34.4%)	
**Size of Incarcerated Inguinal Hernia (cm)**				0.727			0.494
< 5*5	168 (65.6%)	127 (66.5%)	41 (63.1%)		47 (61.8%)	121 (67.2%)	
≥ 5*5	88 (34.4%)	64 (33.5%)	24 (36.9%)		29 (38.2%)	59 (32.8%)	
**Pneumoperitoneum**				0.001			0.635
No	251 (98.0%)	191 (100%)	60 (92.3%)		74 (97.4%)	177 (98.3%)	
Yes	5 (1.95%)	0 (0.00%)	5 (7.69%)		2 (2.63%)	3 (1.67%)	
**Rebound Tenderness**				< 0.001			0.723
No	230 (89.8%)	183 (95.8%)	47 (72.3%)		67 (88.2%)	163 (90.6%)	
Yes	26 (10.2%)	8 (4.19%)	18 (27.7%)		9 (11.8%)	17 (9.44%)	
**Board like Rigidity of the Aabdomen**				< 0.001			0.895
No	235 (91.8%)	185 (96.9%)	50 (76.9%)		69 (90.8%)	166 (92.2%)	
Yes	21 (8.20%)	6 (3.14%)	15 (23.1%)		7 (9.21%)	14 (7.78%)	
**Hernia Sac Effusion**				< 0.001			1.000
No	190 (74.2%)	182 (95.3%)	8 (12.3%)		56 (73.7%)	134 (74.4%)	
Yes	66 (25.8%)	9 (4.71%)	57 (87.7%)		20 (26.3%)	46 (25.6%)	
**Abdominal effusion**				< 0.001			0.216
No	221 (86.3%)	189 (99.0%)	32 (49.2%)		62 (81.6%)	159 (88.3%)	
Yes	35 (13.7%)	2 (1.05%)	33 (50.8%)		14 (18.4%)	21 (11.7%)	
**Surgical Procedure**				0.001			0.571
Open	212 (82.8%)	149 (78.0%)	63 (96.9%)		65 (85.5%)	147 (81.7%)	
Laporoscopy	44 (17.2%)	42 (22.0%)	2 (3.08%)		11 (14.5%)	33 (18.3%)	
**Incision Site**				< 0.001			0.214
Inguinal incision	215 (84.0%)	180 (94.2%)	35 (53.8%)		60 (78.9%)	155 (86.1%)	
Abdominal incision	41 (16.0%)	11 (5.76%)	30 (46.2%)		16 (21.1%)	25 (13.9%)	
**Hernia Mesh**				< 0.001			0.587
No	173 (67.6%)	112 (58.6%)	61 (93.8%)		49 (64.5%)	124 (68.9%)	
Yes	83 (32.4%)	79 (41.4%)	4 (6.15%)		27 (35.5%)	56 (31.1%)	
**White Blood Cell (10**^**9**^**/l)**				< 0.001			0.255
≤ 10	177 (69.1%)	145 (75.9%)	32 (49.2%)		49 (64.5%)	128 (71.1%)	
10 < White Blood Cell < 20	67 (26.2%)	45 (23.6%)	22 (33.8%)		21 (27.6%)	46 (25.6%)	
≥ 20	12 (4.69%)	1 (0.52%)	11 (16.9%)		6 (7.89%)	6 (3.33%)	
**Hemoglobin (g/l)**				0.613			0.207
< 130	125 (48.8%)	91 (47.6%)	34 (52.3%)		32 (42.1%)	93 (51.7%)	
≥ 130	131 (51.2%)	100 (52.4%)	31 (47.7%)		44 (57.9%)	87 (48.3%)	
**Platelet (10**^**9**^**/l)**				0.025			0.419
< 300	206 (80.5%)	147 (77.0%)	59 (90.8%)		64 (84.2%)	142 (78.9%)	
≥ 300	50 (19.5%)	44 (23.0%)	6 (9.23%)		12 (15.8%)	38 (21.1%)	
**Procalcitonin (g/l)**				< 0.001			0.958
< 0.05	183 (71.5%)	170 (89.0%)	13 (20.0%)		55 (72.4%)	128 (71.1%)	
≥ 0.05	73 (28.5%)	21 (11.0%)	52 (80.0%)		21 (27.6%)	52 (28.9%)	
**Neutrophil Ratio (%)**				< 0.001			0.270
< 90	223 (87.1%)	179 (93.7%)	44 (67.7%)		63 (82.9%)	160 (88.9%)	
≥ 90	33 (12.9%)	12 (6.28%)	21 (32.3%)		13 (17.1%)	20 (11.1%)	
**Prothrombin Time (s)**				< 0.001			0.471
< 14	175 (68.4%)	146 (76.4%)	29 (44.6%)		49 (64.5%)	126 (70.0%)	
≥ 14	81 (31.6%)	45 (23.6%)	36 (55.4%)		27 (35.5%)	54 (30.0%)	
**Fibrinogen Concentration (g/l)**				< 0.001			1.000
< 4	193 (75.4%)	155 (81.2%)	38 (58.5%)		57 (75.0%)	136 (75.6%)	
≥ 4	63 (24.6%)	36 (18.8%)	27 (41.5%)		19 (25.0%)	44 (24.4%)	

### Nomogram construction

The association between clinical variables and intestinal necrosis patients was evaluated using univariate analysis. Fisher’s exact test or chi-square test was employed for the analysis of categorical variables. Analysis of variance and Kruskal–Wallis H test were used to compare differences among the three groups.Multivariable logistic regression was applied to select independent predictive factors from the variables that showed significant differences in univariate analysis. The glmnet package was used to run LASSO (Least Absolute Shrinkage and Selection Operator) regression analysis, a shrinkage and variable selection method for linear regression models, with the best lambda value selected. Lambda.1se provided a model with good performance and minimal independent variables. We then conducted logistic regression analysis using the rms package in R software, incorporating the selected features from the LASSO regression model, to construct a predictive model [8].

### Nomogram performance validation

In the external validation process of the nomogram, the total score for each patient in the validation set was calculated based on the established nomogram.Receiver Operating Characteristic (ROC) curve analysis, calibration curve analysis, and Decision Curve Analysis (DCA) were used to estimate the performance of the risk prediction model using data from the training and validation sets, respectively. The pROC package in R software was utilized for ROC curve analysis to calculate the Area Under the Curve (AUC) [9]. The rms package was used to plot and calculate calibration curves to assess the calibration of the flowchart in this study, and the Hosmer–Lemeshow test was performed. Furthermore, the nricens package was used for Decision Curve Analysis (DCA) to determine the net benefits of the nomogram in this study at different threshold probabilities [10].

### Statistical analysis

Statistical analysis was performed using R software (version 3.6.1; http://www.Rproject.org; R Foundation, Vienna, Austria). Fisher’s exact test or chi-square test was used for the analysis of categorical variables. Multivariable logistic regression was applied to select independent predictive factors from the variables that showed significant differences in univariate analysis. The glmnet package was used for LASSO regression analysis. The pROC package in R software was employed for ROC curve analysis. The rms package was used to plot and calculate calibration curves to assess the calibration of the flowchart in this study, and the Hosmer–Lemeshow test was performed. Additionally, the nricens package was used for Decision Curve Analysis (DCA).

## Results

### Characteristics of the study

This study ultimately included a cohort of 256 patients. All patients were divided into training set (*n* = 180) and validation set (*n* = 76) in a ratio of 7:3. Table 1 presents the baseline characteristics of patients with incarcerated inguinal hernia in the training and validation sets, with no significant differences observed between the two groups (*p* > 0.05). Table 1 also categorizes the patients into the group with incarcerated inguinal hernia accompanied by intestinal necrosis (*n* = 65) and the group with incarcerated inguinal hernia without intestinal necrosis (*n* = 191).

### Independent risk factors of the training set

Univariate analysis revealed that factors such as Gender, Age, Cerebral Infarction, Duration of Incarcerated Inguinal Hernia, Rebound Tenderness, Board-like Rigidity of the Abdomen, Hernia Sac Effusion, Abdominal effusion, Surgical Procedure, Incision Site, Hernia Mesh, White Blood Cell 2, Procalcitonin, Neutrophil Ratio, Prothrombin Time, and Fibrinogen Concentration, among others, contribute to the risk profile of patients with incarcerated inguinal hernia accompanied by intestinal necrosis (Table 2).

**Table 2:** Univariate and multivariate analysis of incarcerated inguinal hernia in training cohort

Variables	Uni-B	Uni-SE	Uning-OR	Uni-CI	Uni-P	Multi-B	Multi-SE	Multi-OR	Multi-CI	Multi-P
Fibrinogen Concentration	1.501	0.37871	4.486	4.486(2.142–9.512)	0					
Prothrombin Time	1.179	0.36301	3.25	3.25(1.598–6.667)	0.001					
Neutrophil Ratio	1.548	0.4901	4.704	4.704(1.804–12.59)	0.002					
Procalcitonin	3.304	0.45487	27.222	27.22(11.62–69.98)	0	4.109	1.07159	60.893	60.89(10.99–1143.)	0
Platelet	-0.916	0.51541	0.4	0.4(0.13–1.019)	0.076					
Hemoglobin	-0.648	0.35733	0.523	0.523(0.255–1.043)	0.07					
White Blood Cell 1	0.74	0.38757	2.097	2.097(0.969–4.466)	0.056					
White Blood Cell 2	3.076	1.11861	21.667	21.66(3.3–425.4)	0.006					
Hernia Mesh	-2.627	0.74466	0.072	0.072(0.011–0.249)	0					
Incision Site	2.546	0.4946	12.758	12.75(5.03–35.77)	0					
Surgical Procedure	-2.583	1.03156	0.076	0.076(0.004–0.369)	0.012					
Abdominal effusion	4.723	1.04829	112.5	112.5(21.88–2066.)	0	1.891	1.24655	6.624	6.624(0.744–150.2)	0.129
Hernia Sac Effusion	4.618	0.57075	101.333	101.3(35.68–340.9)	0	4.804	1.10212	122.025	122.0(20.48–2371.)	0
Board like Rigidity of the Aabdomen	1.908	0.58934	6.737	6.737(2.187–23.13)	0.001					
Rebound Tenderness	1.977	0.54366	7.222	7.222(2.558–22.33)	0					
Pneumoperitoneum	16.765	840.27418	19099086.56	19099(0-NA)	0.984					
Size of Incarcerated Inguinal Hernia	0.212	0.36362	1.236	1.236(0.597–2.502)	0.56					
Location of Incarcerated Inguinal Hernia	-0.295	0.37561	0.745	0.745(0.347–1.529)	0.432					
Duration of Incarcerated Inguinal Hernia	1.995	0.40422	7.353	7.353(3.437–16.97)	0					
Cerebral Infarction	1.411	0.58672	4.099	4.099(1.287–13.45)	0.016					
Diabetes	0.461	0.72944	1.585	1.585(0.323–6.294)	0.528					
Coronary Heart Disease	-1.03	0.77111	0.357	0.357(0.055–1.327)	0.182					
Hypertension	0.269	0.38625	1.309	1.309(0.6–2.755)	0.486					
Age	1.911	0.62524	6.758	6.758(2.293–28.96)	0.002					
Gender	-0.933	0.37692	0.393	0.393(0.188–0.83)	0.013					

**Fig. 2:**
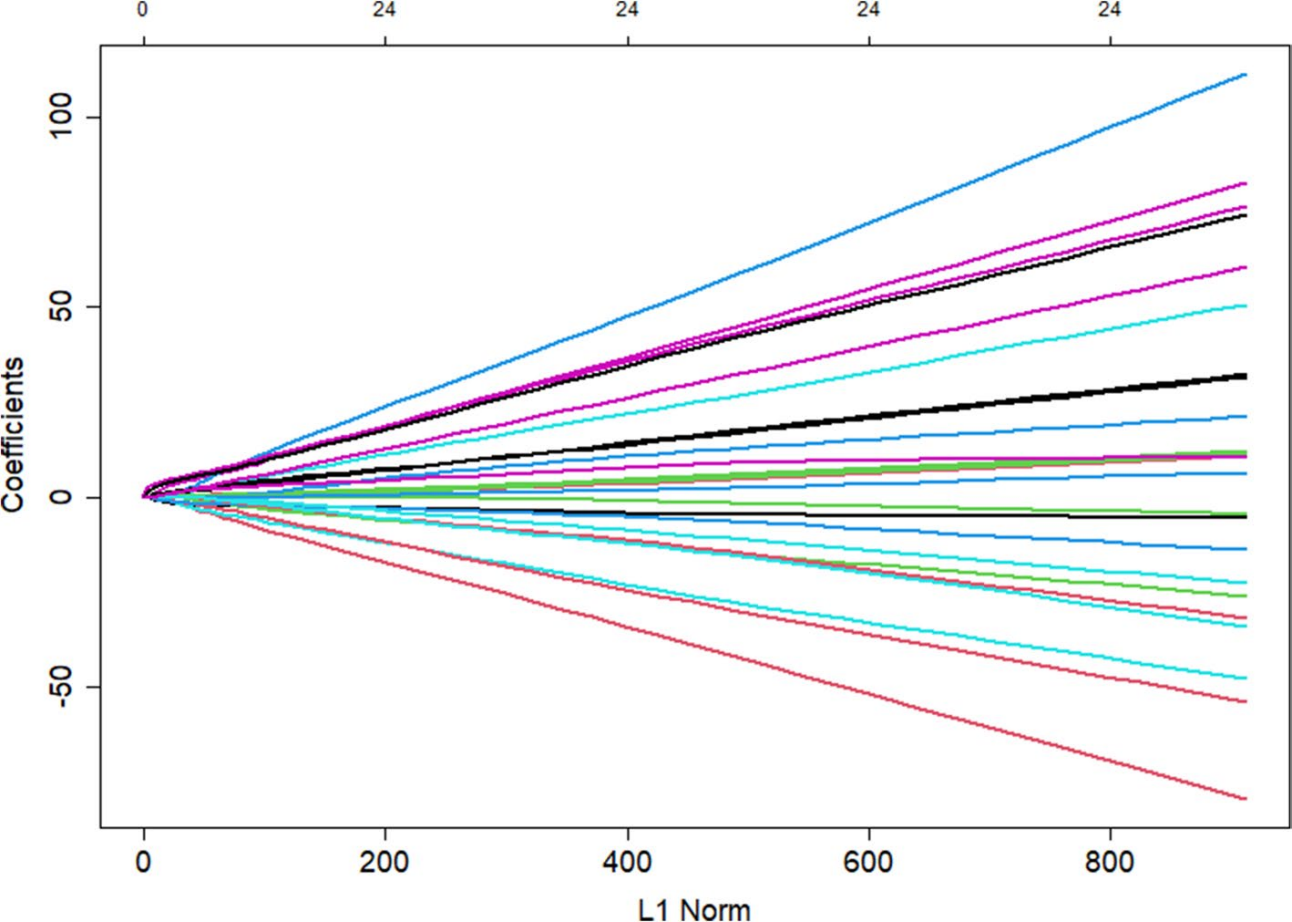
Lasso1

**Fig. 3:**
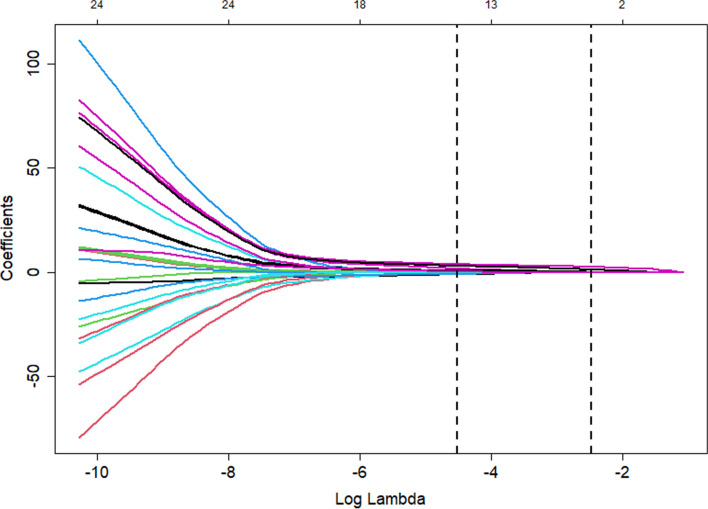
Lasso2

**Fig. 4:**
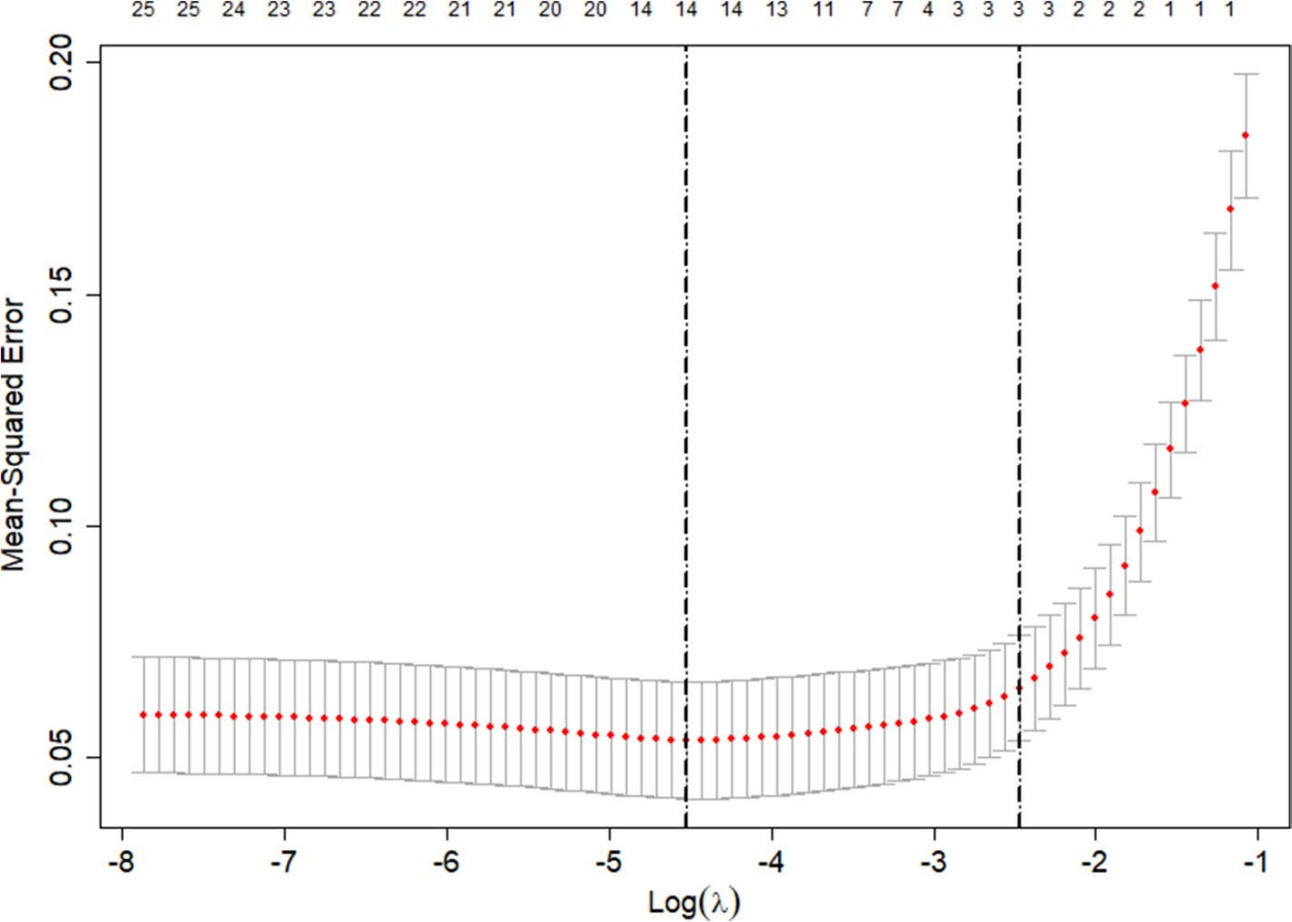
Lambda.1se

Variable selection and model complexity adjustment were performed using the LASSO binary logistic regression model. Figures 2 and 3 demonstrated the optimal lambda value, which corresponded to a model with fourteen non-zero coefficients. The Lambda.1se identified a model with similar predictive capabilities consisting of only three variables (Fig. 4).

### Creation of the predictive model

**Fig. 5:**
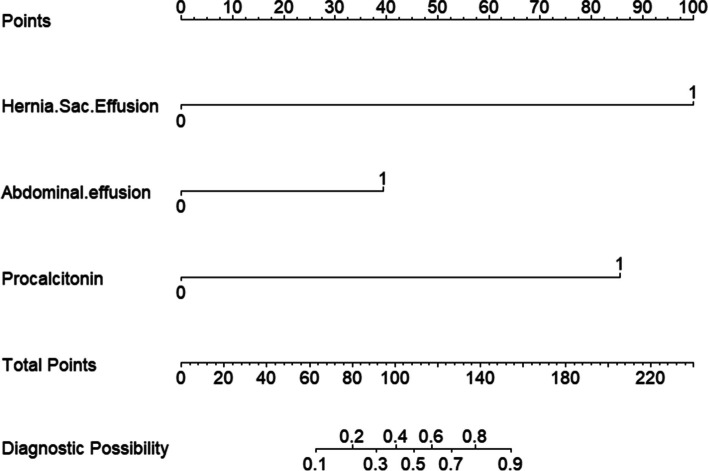
Nomogram graph

Based on the results of the LASSO regression analysis, a multivariate logistic regression model was developed to establish the predictive model. Out of the original 24 variables, three variables, namely Abdominal effusion, Hernia Sac Effusion, and Procalcitonin, were included as predictors in the model. The predictive model was represented by a formal graph, quantifying the risk probability of intestinal necrosis in patients with incarcerated inguinal hernia (Fig. 5).

### Predictive model validation

**Fig. 6:**
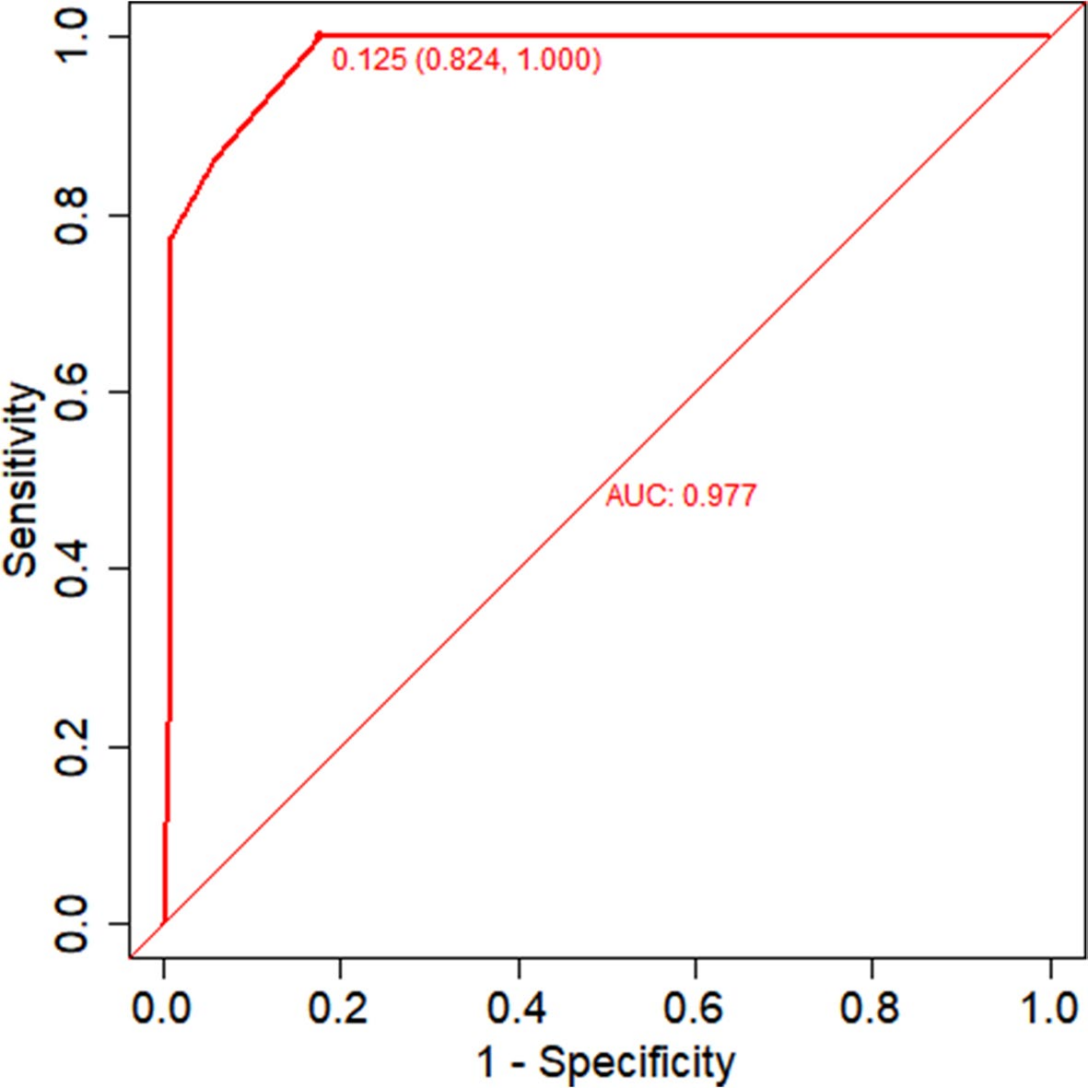
ROC Curve (AUC) for the nomogram graph in the training set

**Fig. 7:**
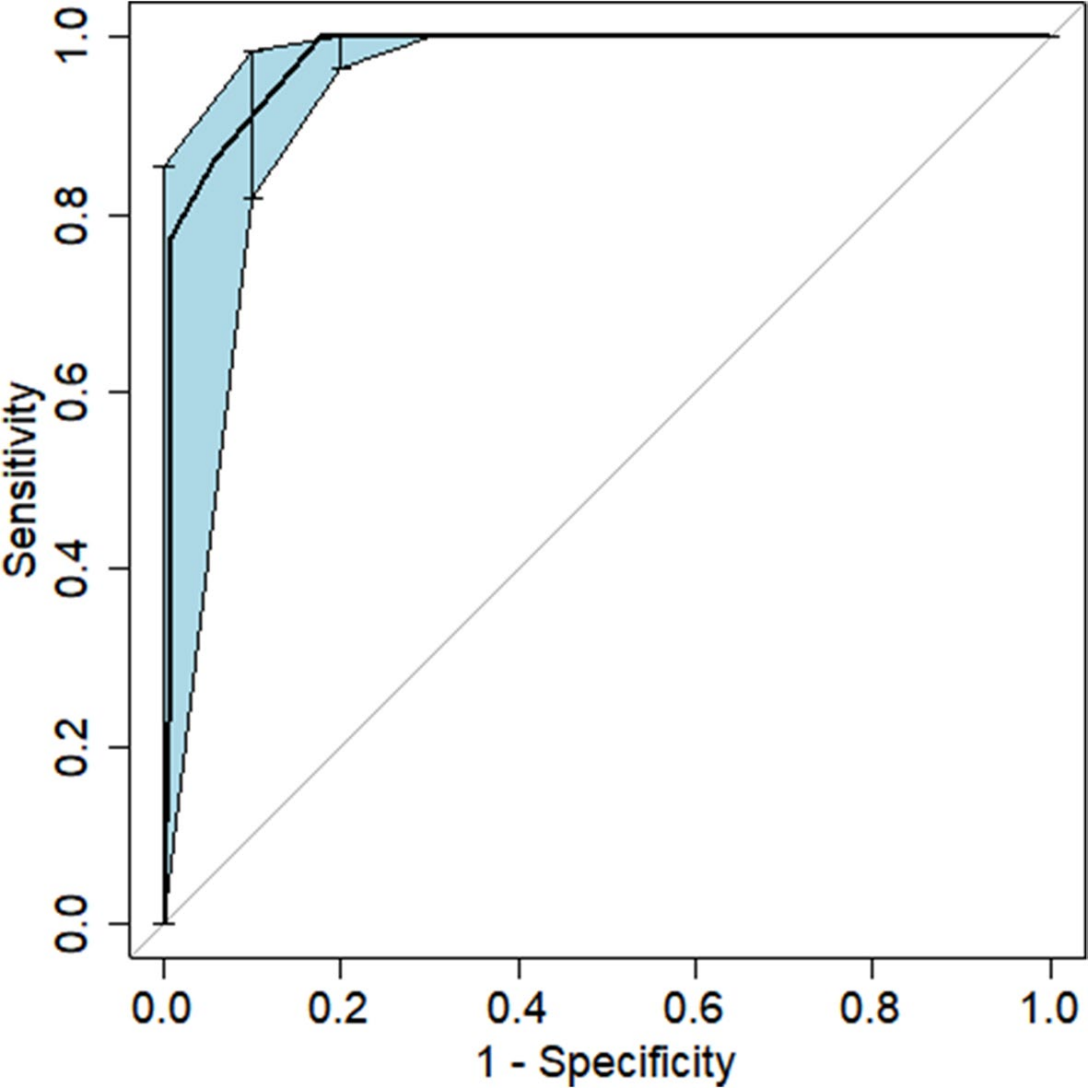
Vertify the Accuracy of the nomogram graph

**Fig. 8:**
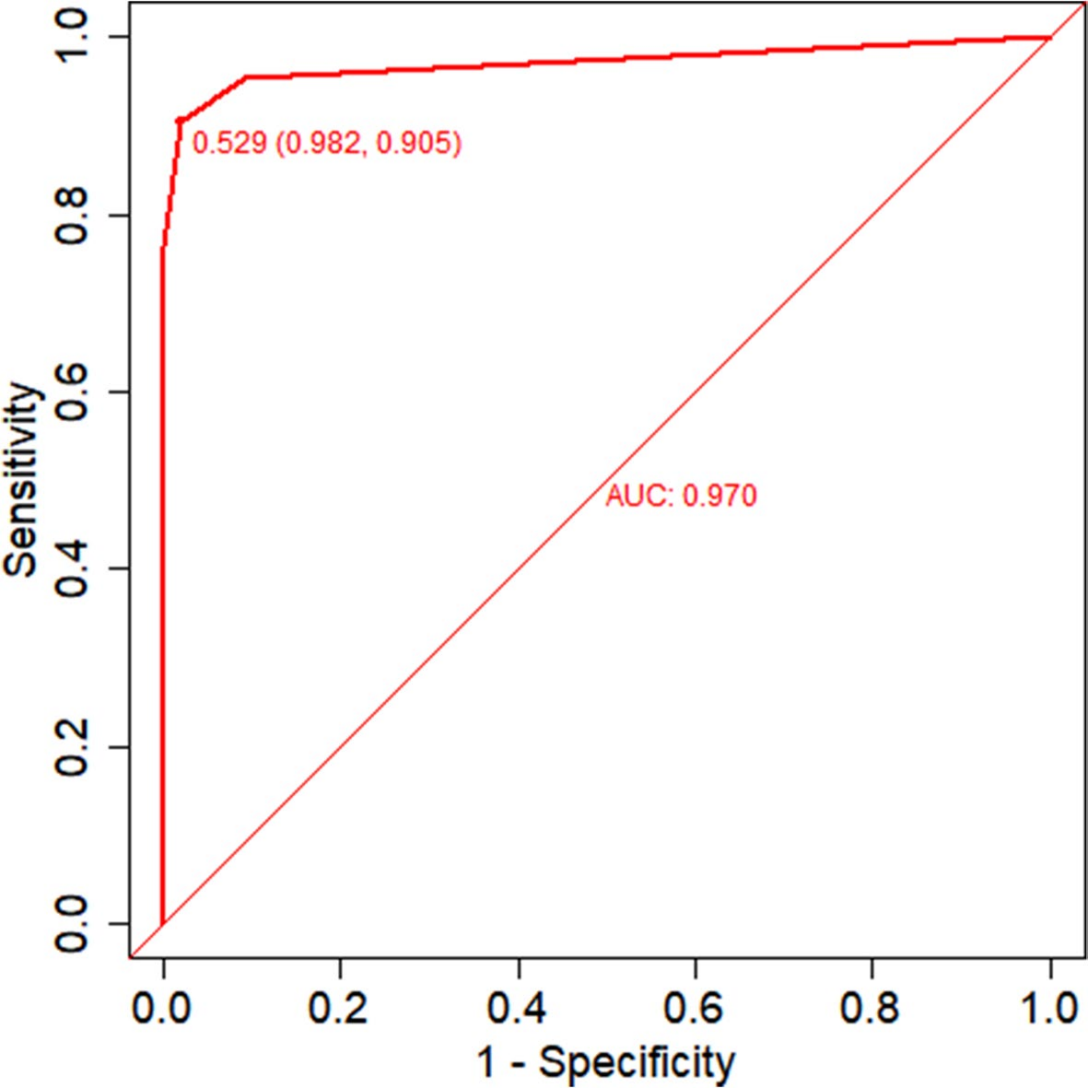
ROC Curve (AUC) for the nomogram graph in the training set

**Fig. 9:**
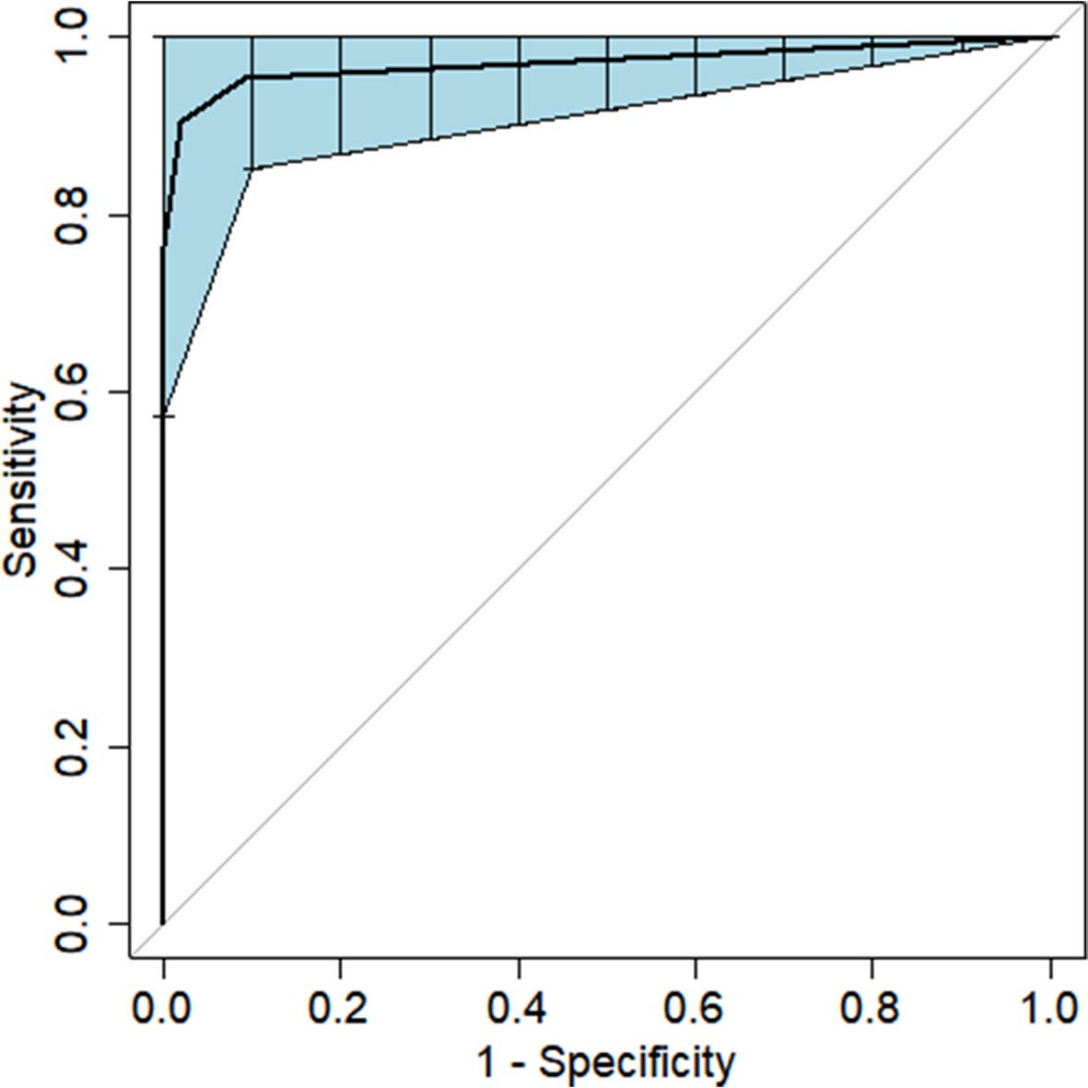
Vertify the accuracy of the nomogram graph

The area under the ROC curve (AUC) for the nomogram graph in the training set was 0.977 (95% CI = 0.957–0.992) (Fig. 6). The accuracy of the nomogram graph was verified using the bootstrap method with 500 resamplings (Fig. 7). In the validation set, the AUC for the nomogram graph was 0.970 (95% CI = 0.91–1) (Fig. 8), and its accuracy was assessed using the bootstrap method with 500 resamplings (Fig. 9). These findings indicate excellent discriminatory performance of the discovered nomogram model.

**Fig. 10:**
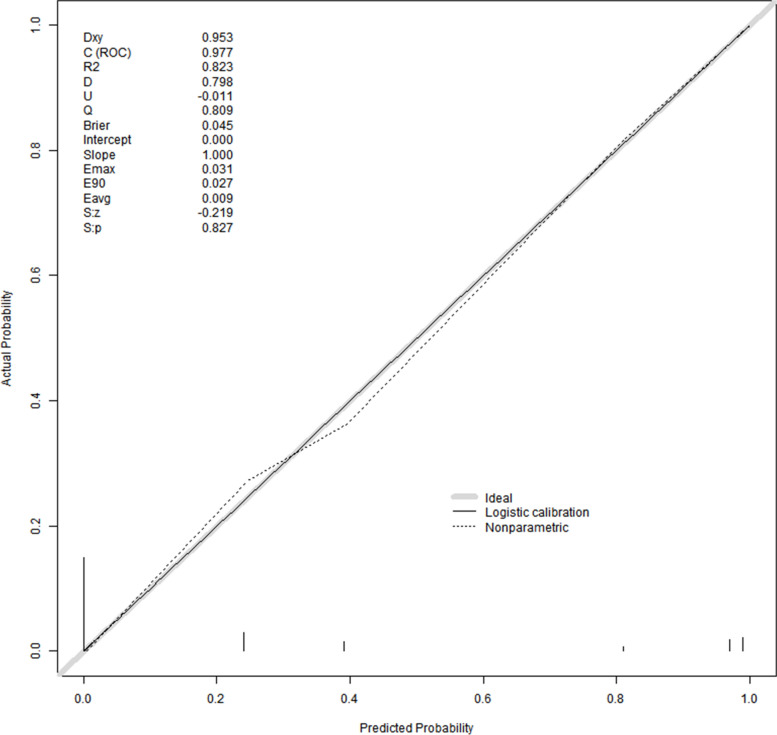
The calibration curves of the predictive model

**Fig. 11:**
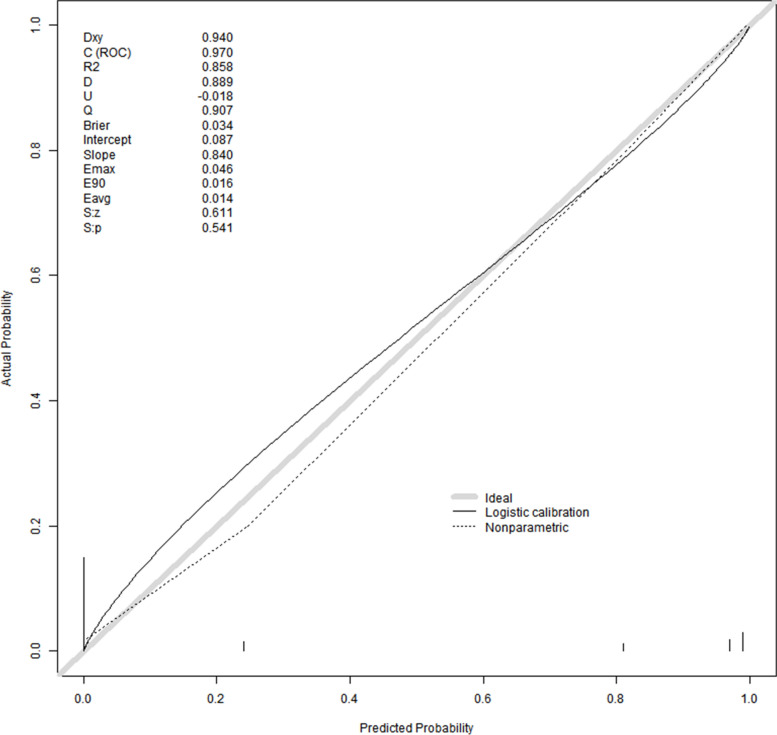
The calibration curves of the validation set

To assess the calibration of the predictive model, calibration curves and the Hosmer–Lemeshow test were employed. The calibration curves (Figs. 10 and 11) illustrate a strong fit between the predictive model and the validation set. The Hosmer–Lemeshow test revealed high consistency between the predicted and actual probabilities (training set, *P* = 0.053; validation set, *P* = 0.990).

**Fig. 12:**
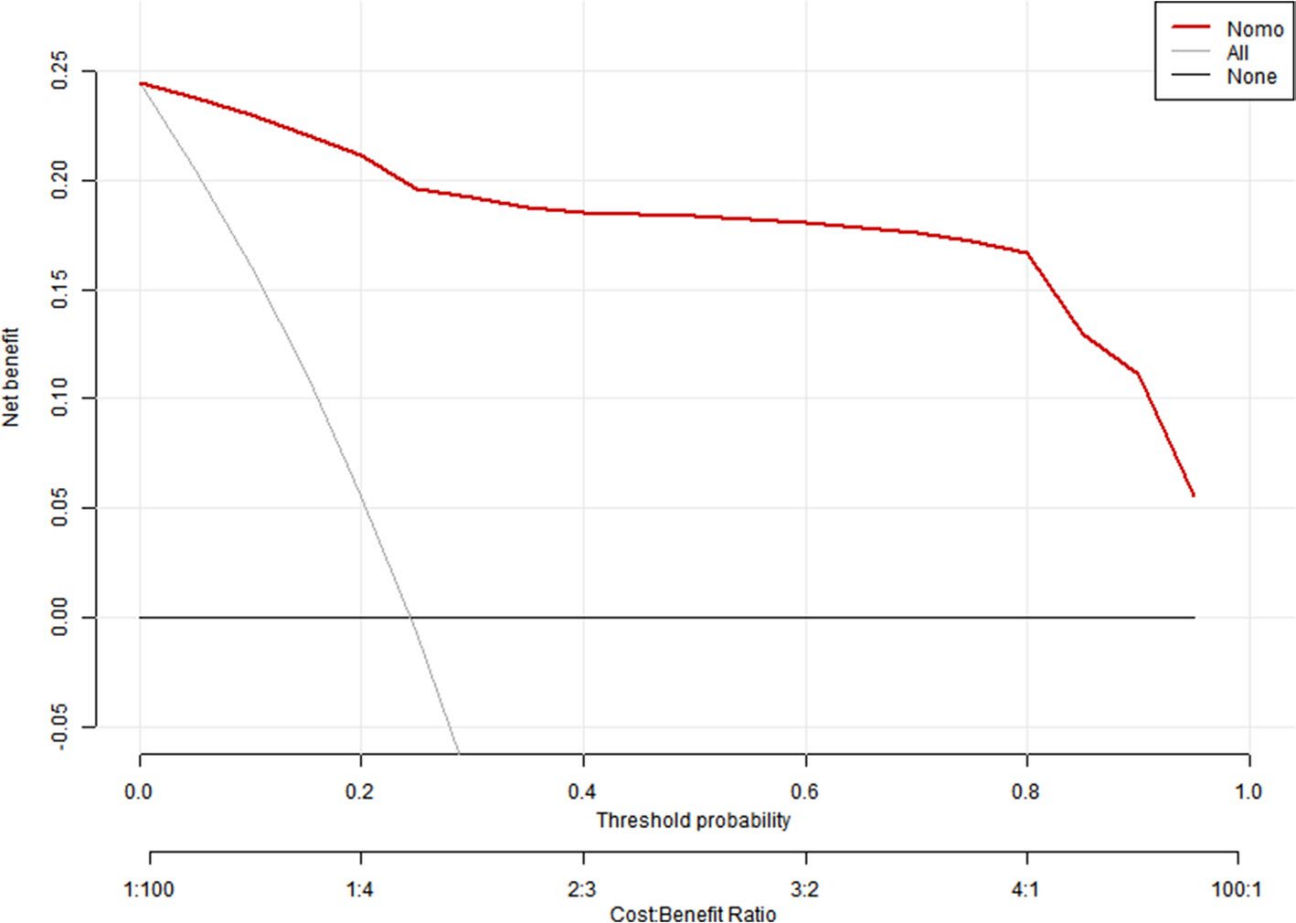
The decision curve analysis of the training sets

**Fig. 13:**
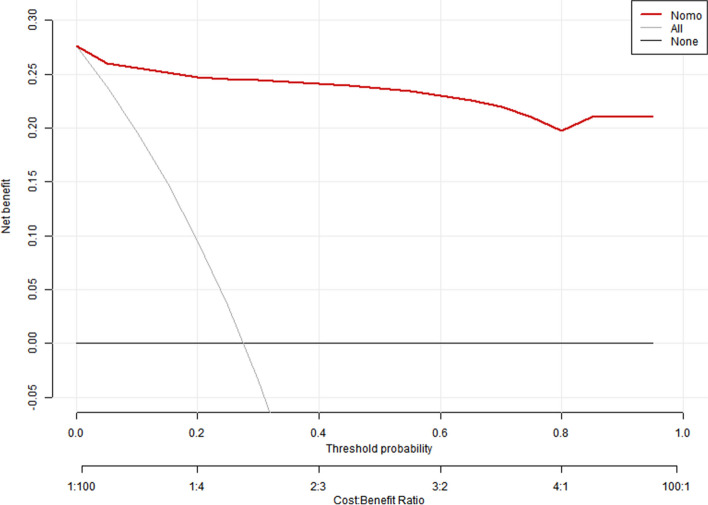
The decision curve analysis of the validation sets

The Decision Curve Analysis (DCA) illustrated that patients could derive net benefit when the threshold probability of the training and validation sets ranged from 0 to 100% (Figs. 12 and 13).

## Discussion

Incarcerated inguinal hernia (IGH) is a common surgical abdominal emergency, accounting for 50–80% of incarcerated abdominal wall hernias. Approximately 15% of IGH patients required intestinal resection due to associated intestinal necrosis [11]. The postoperative complication rate of emergency surgery for IGH was reported to be 21–39%, with a mortality rate of 4–5% [12]. Early identification of predictive factors for incarcerated inguinal hernia accompanied by intestinal necrosis and prompt surgical intervention held significant clinical significance.

Currently, research on postoperative complications and prognosis following inguinal hernia incarceration is fairly extensive. However, studies assessing the occurrence of intestinal necrosis in patients with incarcerated inguinal hernias preoperatively are relatively scarce. Furthermore, there is a paucity of relevant research establishing predictive models based on limited emergency patient information. In light of these circumstances, this study aimed to establish a predictive model through a retrospective approach to assist surgeons in accurately assessing the risk of intestinal necrosis in patients preoperatively. Through this predictive model, we aspired to furnish surgeons with more precise preoperative information, enhancing the selection of the optimal timing for surgery and the determination of the appropriate surgical approach, thereby maximizing the prognosis for patients. We firmly believed that through this research, we could offer robust support for preoperative diagnosis and treatment decisions in patients with incarcerated inguinal hernias, bringing about substantive improvements in clinical management for the benefit of the patients.

Previously, multiple studies indicated that factors such as female gender, advanced age, prolonged hospitalization, inguinal hernia, and peritonitis contributed to the risk of incarcerated inguinal hernia leading to intestinal necrosis. However, these studies exhibited contradictory findings. For instance, Abd Ellatif et al.’s research [6] suggested that female gender was not a risk factor for bowel resection, while other studies [7, 8] indicated that it was indeed a risk factor. Moreover, previous studies had predominantly relied on small sample sizes. Hence, there was an urgent need for a comprehensive study to early identify the risk factors for bowel resection in patients with incarcerated inguinal hernia complicated by intestinal necrosis.

The management of incarcerated groin hernias remained a subject of contention. While the majority of scholars emphasized prompt surgical intervention, some researchers proposed, in selectively chosen cases, employing hernia reduction under analgesia and sedation conditions [13]. Early and accurate identification of intestinal necrosis was paramount for formulating timely surgical treatment plans when dealing with hernia patients [14]. Delay in diagnosis and surgical intervention might have resulted in a higher rate of bowel resection, concurrently increasing postoperative morbidity and mortality [15]. Hence, in predicting and diagnosing cases of intestinal necrosis in patients with an Incarcerated groin hernia through this model, foremost consideration should be given to surgical intervention; in this context, the reduction of the hernia should be explicitly defined as a contraindication for treatment. According to the surgical treatment guidelines [16], if the patient was devoid of intestinal necrosis or harbors intestinal necrosis without enteric fluid leakage (clean-contaminated surgical field, CDC wound class II), a tension-free inguinal hernia repair procedure may be pursued. However, in the presence of both intestinal necrosis and associated enteric fluid leakage (contaminated surgical field, CDC wound class III), the patient was constrained to undergo a high ligation procedure for the hernia sac.

Our study delineated that initially, 21 patients manifested with board-like abdominal symptoms. However, intraoperative validation subsequently disclosed that only 15 among them received a definitive diagnosis of intestinal necrosis. Conversely, within the cohort of 235 patients who lacked board-like abdominal symptoms prior to the surgical procedure, 50 were unequivocally identified as harboring intestinal necrosis. This underscores that, while peritonitis serves as a robust predictor of bowel ischemia, its absence does not entirely preclude the potential emergence of this pathological condition.The primary objective underlying the design of our model is to prognosticate a subset of patients characterized by the presence of intestinal necrosis but lacking the manifestation of peritonitis symptoms. By employing our model, one should be able to anticipate this specific subset devoid of peritonitis yet affected by intestinal necrosis.

This study revealed that Abdominal effusion, Hernia Sac Effusion, and Procalcitonin were three primary variable factors influencing the occurrence of bowel necrosis in patients with incarcerated inguinal hernia. When the intestinal tube was compressed by the hernia sac, causing impaired blood supply, leakage of fluid occurred in the intestines and mesentery. This further progressed to intestinal necrosis, leading to the accumulation of bloody intraperitoneal fluid. However, in clinical surgery, the presence of bloody intraperitoneal fluid did not necessarily indicate bowel necrosis; it may simply indicate compromised blood flow in the intestines. As long as the incarcerated intestinal tube was promptly released, normal intestinal function could be restored. Procalcitonin (PCT) [17], primarily secreted by thyroid C cells, was a protein containing a 116-amino acid sequence. Under physiological conditions, serum procalcitonin levels were typically very low, usually < 0.1 ng/ml. PCT could serve as a marker for infection and sepsis, and studies had shown that when serum PCT levels exceeded 1.0 ng/ml, the occurrence of bowel necrosis should be highly suspected [18]. Our study found significant differences between two patient groups when stratifying procalcitonin at a cutoff of 0.05 ng/ml. Furthermore, when constructing a predictive model using the risk factors of Abdominal effusion, Hernia Sac Effusion, and Procalcitonin > 0.05 ng/ml, the model demonstrated excellent discrimination performance with an area under the ROC curve (AUC) of 0.977 for the training set and an AUC of 0.970 for the validation set. The calibration curve (Fig. 6) indicated a very good fit between the predictive model and the validation set. The DCA curve demonstrated that using this research model provided net benefits for patients.

In our study, the presence of hernia sac or abdominal cavity fluid was considered conclusive, regardless of the quantity, if detected through preoperative abdominal ultrasound or abdominal CT examinations. We refrained from further stratified analysis of hernia sac or abdominal cavity fluid because the model’s predictive capability exhibited excellent performance merely by incorporating the presence or absence of hernia sac or abdominal cavity fluid as model parameters, with an AUC reaching 0.97. This straightforward yet effective approach provided us with a feasible means of preoperative diagnosis in clinical practice, simultaneously demonstrating the outstanding performance of the model in predicting the risk of intestinal necrosis in patients.

When inguinal hernia progressed to incarcerated inguinal hernia, inflammatory markers such as white blood cell count and percentage of neutrophils increased [19]. However, the degree to which increased inflammatory markers could indicate a high risk of bowel necrosis remained uncertain. Our study divided white blood cell count into three groups using 10^9^/L and 20^9^/L as cutoffs and divided the neutrophil ratio into two groups based on 90%. Univariate analysis showed differences between the groups, but multivariate analysis revealed no statistically significant differences between the groups, indicating that they could not serve as predictive indicators for bowel necrosis. Despite previous studies [4, 20] indicating an association between white blood cell count and the percentage of neutrophils with the occurrence of intestinal necrosis in incarcerated hernia patients, our research, under a multifactorial analytical framework, revealed that these two biological markers did not exhibit significant predictive roles for intestinal necrosis in our established model, in contrast to other variables we meticulously selected.

Currently, the literature [21–24] on factors influencing intestinal necrosis in incarcerated inguinal hernias is abundant. However, research on diagnostic models predicting the occurrence of intestinal necrosis in patients with incarcerated inguinal hernias is relatively sparse. To address this gap in the research domain, this study adopted a systematic and comprehensive approach, constructing a predictive model focusing on three key variables.

The selection of these three key variables was meticulously considered. They not only facilitated straightforward preoperative examinations but could also be easily obtained through preoperative serological tests and abdominal ultrasound examinations. The design of this strategy aimed to ensure the practicality and broad applicability of the model, making it more readily applicable in clinical practice.

The model exhibited remarkable performance in evaluations, with an AUC reaching 0.97, surpassing the widely recognized discriminative ability threshold of 0.95 [25]. This signified that our model not only achieved significant prowess in predictive capability but also held promising prospects for highly credible application in clinical practice.

The significance of this study lay not only in filling research gaps in relevant fields but also in providing surgeons with a practical guiding tool. We firmly believed that this model was poised to play a positive role in clinical work, offering robust support for early prediction and intervention of intestinal necrosis in incarcerated inguinal hernias. Consequently, it had the potential to enhance treatment outcomes and survival rates for patients.

This study established a novel, convenient, and highly accurate nomogram [26] for predicting the probability of bowel necrosis occurrence in patients with incarcerated inguinal hernia. The nomogram demonstrated satisfactory consistency in both the modeling population and the validation population, indicating its excellent clinical applicability. Nomograms [27] are widely used for predicting the incidence of various diseases or complications, providing references for clinical decision-making. They also meet the demands of integrating biological and clinical models and pursuing personalized medicine, thus providing individualized predictions for patients.

In conclusion, bowel necrosis in patients with incarcerated inguinal hernia was caused by multiple factors. The nomogram predictive model constructed in this study could be used to predict and differentiate whether incarcerated inguinal hernia patients would experience bowel necrosis. By promptly implementing reduction techniques or surgical interventions, the risk of bowel necrosis in such patients could be reduced. This held significant clinical importance in improving patient prognosis and enhancing their quality of life. However, this study also had some limitations: Firstly, due to the emergency nature of the subjects, some crucial indicators such as lactate levels, erythrocyte sedimentation rate, and C-reactive protein were unattainable. This limitation might have affected the comprehensive assessment of patient conditions and the synthesis of research outcomes. Secondly, the radiological manifestations of intestinal necrosis are diverse [16]. However, considering the simplicity of detection indicators and the practical feasibility of the model, we opted to focus solely on the examination of abdominal and hernia sac fluid accumulation for in-depth investigation. Although these two indicators were relatively easy to obtain in clinical practice, to some extent, this approach might have overlooked other significant radiological features that could have influenced intestinal necrosis. Thirdly,The research data was derived from a retrospective study in a single center, which had certain limitations, and the relevant conclusions needed further validation through large-scale prospective studies.

### Supplementary Information

**Additional file 1.**

## Data Availability

All relevant data were within the manuscript and its Additional files.

## Abbreviations

IH: Inguinal Hernia
IIH: Incarcerated Inguinal Hernia
BN: Bowel Necrosis
LASSO: Least Absolute Shrinkage and Selection Operator
DCA: Decision Curve Analysis
ROC: Receiver Operating Characteristic
